# HC-SPA: Hyperbolic Cosine-Based Symplectic Phase Alignment for Fusion Optimization

**DOI:** 10.3390/s25165003

**Published:** 2025-08-13

**Authors:** Wenlong Zhang, Aiqing Fang, Ying Li, Yan Wei

**Affiliations:** 1 College of Computer and Information Science, Chongqing Normal University, Chongqing 401331, China; 2023210516099@stu.cqnu.edu.cn (W.Z.); aiqingf@mail.nwpu.edu.cn (A.F.); 2 Ningbo Research Institute of Northwestern Polytechnical University, Xi’an 710072, China; liying_npu@mail.nwpu.edu.cn

**Keywords:** multimodal fusion, gradient optimization, hyperbolic geometry, symplectic structure

## Abstract

In multimodal collaborative learning, the gradient dynamics of heterogeneous modalities face significant challenges due to the curvature heterogeneity of parameter manifolds and mismatches in phase evolution. Traditional Euclidean optimization methods struggle to capture the complex interdependencies between heterogeneous modalities on non-Euclidean or geometrically inconsistent parameter manifolds. Furthermore, static alignment strategies often fail to suppress bifurcations and oscillatory behaviors in high-dimensional gradient flows, leading to unstable optimization trajectories across modalities. To address these issues, inspired by hyperbolic geometry and symplectic structures, this paper proposes the Hyperbolic Cosine-Based Symplectic Phase Alignment (HC-SPA) fusion optimization framework. The proposed approach leverages the geometric properties of hyperbolic space to coordinate gradient flows between modalities, aligns gradient update directions through a phase synchronization mechanism, and dynamically adjusts the optimization step size to adapt to manifold curvature. Experimental results on public fusion and semantic segmentation datasets demonstrate that HC-SPA significantly improves multimodal fusion performance and optimization stability, providing a new optimization perspective for complex multimodal tasks.

## 1. Introduction

Multimodal fusion refers to the process of comprehensively utilizing information from different modal sensors or data sources and integrating the advantageous features of each modality through specific algorithms to form a more comprehensive, accurate, and robust representation, thereby enhancing the system’s performance in autonomous driving scenarios [1,2]. A prime example is the fusion of infrared and visible images [3], which significantly bolsters system robustness in complex environments by integrating texture details and thermal radiation [4]. The intrinsic physical property distinctions between different modalities, where visible images can capture color and texture details [5], while infrared images delineate object temperature based on thermal radiation, result in marked disparities in the extracted features [6]. These disparities encompass variations in geometric properties, distribution characteristics, and scale, which manifest as gradient conflicts between modalities during the training of deep learning models [7]. Such conflicts impede the harmonization of gradient direction and magnitude across different modalities, ultimately leading to optimization instability [8].

Researchers have conducted extensive research on the fusion of infrared and visible images to address the aforementioned challenges. These efforts can be categorized into two main types [9]: structural improvements and optimization modeling of loss functions. The former involves multiscale feature extraction, where deep learning networks (e.g., CNN [10], GAN [11], Transformer [12], and diffusion [13]) equipped with hierarchical architectures (e.g., pyramid networks or atrous spatial pyramid pooling) are employed to capture both fine-grained textures (from visible images) and coarse thermal radiation (from infrared images) at multiple resolutions. This ensures that salient features are preserved across scales, reducing information loss. Beyond structural improvements, optimizing the loss function has been pivotal in guiding fusion models toward generating high-fidelity, task-relevant outputs. Task-specific losses, tailored to downstream applications (e.g., object detection or semantic segmentation), ensure that the fused image retains critical information. For instance, the semantic segmentation-based fusion loss [14], the content perceptual fusion loss [11], and the quality-aware fusion loss [15] have been proposed. By optimizing the loss function to align with both data fidelity and task requirements, researchers have achieved more robust and informative fusion outcomes [16], overcoming the limitations of traditional Euclidean-based models. Although current approaches have improved fusion performance, several critical limitations persist [17,18]: (1) Many solutions are based on the assumption of Euclidean space, where feature representations are assumed to be effectively fused through linear or simple nonlinear transformations. However, the complexity and hierarchical nature of multimodal data often exceed the expressive power of Euclidean space, leading to an inability to fully explore the deep relationships between modalities during the fusion process. (2) These methods typically focus on the design of feature extraction and fusion but overlook the coordination of gradient dynamics during optimization. Due to gradient conflicts and phase mismatches between modalities, the training process may experience oscillations or converge to local optima, reducing learning efficiency and model stability. (3) Many solutions primarily rely on linear or simple nonlinear transformations for feature fusion. However, when dealing with multimodal data that have complex structures and hierarchical relationships, these methods often fail to fully exploit the deep relationships between modalities, resulting in suboptimal fusion performance.

To address the above issues, inspired by hyperbolic geometry [19], this paper propose a hyperbolic cosine-based symplectic phase alignment fusion optimization algorithms. The core innovation lies in projecting the gradients of different modalities into a shared hyperbolic space and utilizing hyperbolic metrics to calculate and coordinate inter-modal gradient flow. This approach leverages geometric properties directly at the parameter optimization level rather than the feature level, thereby more effectively addressing the gradient conflict problem. Additionally, we introduce symplectic transformations to align the phase of gradient updates for each modality, ensuring that optimization steps maintain directional consistency. Based on these insights, we propose the Hyperbolic Cosine-Based Symplectic Phase Alignment (HC-SPA) framework, aiming to enhance multimodal fusion performance by combining geometry and optimization theory. The core contributions of HC-SPA include the following three points:

- We have designed a novel gradient coordination mechanism that projects gradients from different modalities onto a shared hyperbolic space. By utilizing hyperbolic metrics to define and compute the geometric relationships between these modalities, this mechanism effectively coordinates their gradient flows, thereby reducing inter-modality conflicts and enhancing the quality of feature fusion through more cooperative updates.
- We have developed a symplectic phase alignment strategy that employs symplectic transformations to rigorously synchronize the gradient update phases across each modality. This design ensures that optimization steps maintain directional consistency, which in turn avoids destructive interference between disparate gradient influences and significantly improves both the stability and convergence speed of the training process.
- We also demonstrate through experiments on several public datasets and different models that the HC-SPA optimizer significantly enhances model performance across various application scenarios. Particularly in tasks involving complex modal relationships, the optimizer effectively improves gradient coordination, reduces phase conflicts, and thereby enhances training stability and fusion performance.

This paper is structured as follows: Section 2 introduces the relevant works, Section 3 proposes the HC-SPA fusion optimization method, Section 4 details the experimental setup and comparative and ablation studies, and the conclusions and future work are drawn in Section 5.

## 2. Related Work

This section begins with a review of the development of multimodal image fusion and the hyperbolic approaches, followed by an analysis of the challenges and limitations of existing methods.

### 2.1. Multimodal Fusion

Research on multimodal fusion originated from the need to integrate heterogeneous data, particularly in computer vision tasks such as infrared and visible image fusion. Early methods primarily relied on handcrafted feature extraction techniques. For example, researchers like in [20] extracted features from visible images using edge detection and texture analysis, while proposing thermal signal features from infrared images. These features are then fused using simple strategies such as feature vector concatenation [21] or weighted averaging [22] to generate a unified representation. However, these methods showed limited performance in complex scenarios because handcrafted features are unable to capture the dynamic relationships between modalities [23] and the diversity of high-dimensional data. The advent of deep learning significantly advanced the field, enabling the learning of more robust and discriminative features for fusion. Unlike traditional methods, deep learning models can automatically extract hierarchical features from raw data, capturing both low-level details (e.g., edges, textures) and high-level semantics (e.g., objects, scenes). This capability allows for a more effective integration of multimodal information. For instance, Ma et al. [24] introduced the concept of adversarial learning into image fusion, proposing numerous adversarial fusion network frameworks that have, to some extent, improved the fusion quality of multimodal data. Tang et al. [10] combined the image fusion task with downstream semantic segmentation tasks, developing a series of semantic-aware multi-task fusion optimization methods. These approaches effectively balance the objectives of both upstream fusion and downstream semantic analysis tasks. Additionally, to enhance the semantic and quality-aware perception of fusion models, researchers have unified the modeling representation of degradation information with the fusion process, thereby constructing numerous fusion models. For instance, DDcGAN [11] established a strong baseline by using a dual-discriminator GAN to preserve detailed textures and salient targets from both source modalities. To address artifacts arising from poor lighting, DIVFusion [15] specifically designed an architecture to be ‘‘darkness-free,” effectively preventing information loss from degraded visible images. Building upon this, a work by Wang et al. [25] introduced a degradation-aware network that adaptively guides the fusion process based on input image quality, enhancing robustness in real-world scenarios. More recently, the paradigm has shifted towards interactivity, with methods like Text-IF [26] leveraging semantic text prompts to give users fine-grained control over the fusion outcome. While these architectural and interactive innovations are powerful, they largely rely on standard optimizers ill suited for the inherent gradient conflicts in multi-modal learning. Our work diverges from a purely architectural focus to address this fundamental optimization challenge, proposing an optimizer specifically designed to manage these conflicts.

### 2.2. Hyperbolic Approaches

In recent years, hyperbolic geometry has emerged as a powerful tool in deep learning, particularly suited for modeling data exhibiting hierarchical structures or complex relational graphs that are poorly represented by Euclidean space. Hyperbolic spaces offer exponential capacity, allowing them to embed tree-like structures and metric spaces with low distortion using significantly fewer dimensions compared with Euclidean space [27]. This inherent capability aligns well with the characteristics of certain types of multimodal data where features may form natural hierarchies or complex non-linear manifolds. Consequently, research has begun exploring the integration of hyperbolic geometry into deep learning models for tasks involving structured or multimodal data. These efforts often focus on learning hyperbolic embeddings for multimodal features [28], where features from different modalities are mapped into a shared or separate hyperbolic space to leverage its geometric properties for better organization and representation of hierarchical or complex relationships. Other approaches have developed neural network layers operating directly in hyperbolic space (e.g., hyperbolic convolutions, hyperbolic attention) [29], which are then applied to process multimodal features or facilitate fusion by utilizing hyperbolic distances and geometries within attention mechanisms or dedicated fusion layers.

The primary goal of these methods is to leverage the unique geometric properties of hyperbolic space to capture complex dependencies and hierarchical relationships within and between modalities more effectively at the representation level [30]. Despite their success in enhancing feature representation and potentially improving performance on downstream tasks by providing a richer embedding space, these existing hyperbolic approaches for multimodal fusion predominantly focus on the feature embedding or representation layer. A key limitation is that they typically do not extend the benefits of hyperbolic geometry to the crucial model parameter optimization process [31]. This means that the optimization is often still performed using standard Euclidean gradient descent techniques, which may not be optimal for parameters associated with hyperbolic representations or for effectively coordinating gradient updates arising from disparate multimodal signals [32]. Consequently, these methods do not fundamentally address the prevalent gradient conflicts and the need for coordinated optimization dynamics commonly encountered in multimodal learning, potentially limiting their ability to fully capture deep [33], intertwined nonlinear relationships across high-dimensional multimodal data and achieve optimal fusion performance.

Drawing inspiration from the potential of non-Euclidean geometry and the critical need for robust optimization in multimodal learning, we re-examine the fusion problem. We posit that addressing both the geometric nature of data heterogeneity and the dynamic behavior of gradients in parameter space is crucial. Unlike previous works that might apply hyperbolic geometry primarily to feature space, our innovation lies in leveraging hyperbolic geometry to analyze and coordinate gradient dynamics during optimization. We move beyond traditional Euclidean optimization assumptions to devise an optimizer that understands and navigates the complex landscape shaped by multimodal gradients. Furthermore, by incorporating principles from symplectic geometry, we introduce a novel mechanism for phase alignment, ensuring that updates from different modalities are synchronized and constructive. This integrated approach, targeting the optimization process within a hyperbolic-symplectic framework, fundamentally distinguishes our work from existing methods and provides a powerful means to enhance training stability and fusion performance.

## 3. Materials and Methods

This subsection begins by introducing the motivation for the proposed method, followed by a comprehensive overview. Subsequently, each mechanism within the method is described in detail.

### 3.1. Motivations

**Figure 1:**
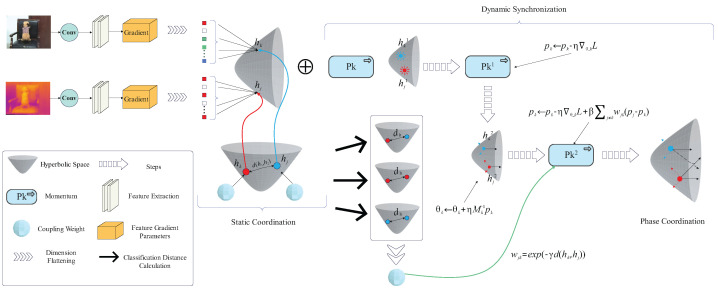
Core schematic diagram of HC-SPA framework.

Visible images primarily capture color and texture details, while infrared images, which rely on thermal radiation, emphasize temperature distribution and structural contours. This heterogeneity results in significant differences in feature representation within the geometric space. Traditional fusion methods typically operate in Euclidean space, integrating features through simple concatenation or weighted fusion. However, this approach often fails to grasp the intricate interplay and the deep, layered patterns that connect the different modalities. As a result, it can lead to the loss of important details or a flawed final combination. To address these challenges, this paper proposes the HC-SPA framework. The proposed method addresses gradient conflicts and training instability in multimodal fusion by integrating geometric deep learning with optimization theory. Figure 1 illustrates the main structure of the HC-SPA framework. First, in the mechanism for coordinating non-uniform gradient flows, it leverages the negative curvature property of hyperbolic geometry to map parameters from Euclidean space into hyperbolic space. This enables the capture of complex nonlinear relationships across modalities. Hyperbolic distances are then used to compute adaptive coupling weights, which modulate momentum updates to coordinate gradient flows in spatial dimensions, reduce conflicts, and establish a stable geometric foundation. Subsequently, inspired by Hamiltonian mechanics, the framework introduces a phase synchronization mechanism. It incorporates momentum variables to simulate the optimization process. Based on loss gradients and a phase coordination term, it synchronizes the rhythm of parameter updates using hyperbolic distances and coupling weights. This not only aligns updates across different agents but also unifies the step sizes within each agent. By preserving the symplectic structure of the phase space, the framework ensures temporal stability and avoids dynamic divergence.

### 3.2. Coordinating Heterogeneous Gradient Flows

#### 3.2.1. Mapping Parameters to the Hyperbolic Space

**Figure 2:**
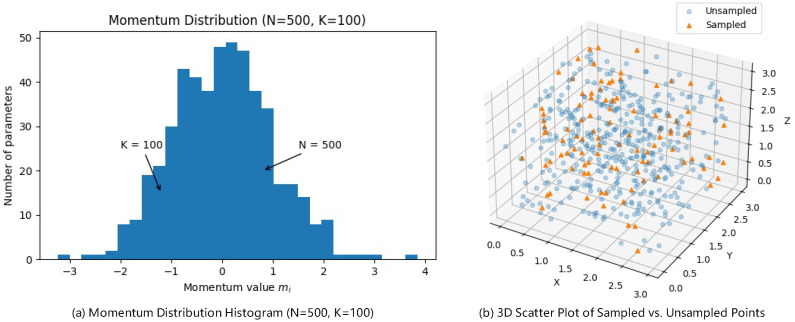
Subset momentum sampling step.

Before performing hyperbolic projection, we can perform a subset momentum sampling step for the optimizer based on the size of the parameter count, we first compute the momentum estimation for all *N* parameters in the Euclidean parameter space, as shown in Figure 2, and assume that its statistical distribution approximately follows a normal distribution with a mean of 0 and a variance of 1. Then, we randomly select a subset of size *K* (where K<N). Next, we map these parameters onto the hyperbolic space manifold used by the optimizer. We then perform the computationally intensive, curvature-aware update only on the *K* sampled points. This involves adjusting the learning rate and direction using the hyperbolic metric tensor to move more precisely along the optimal path defined by the space’s geometry. For the remaining N−K parameters, we use first-order momentum or a conventional model update to keep the computation lightweight. The core reason for this approach is twofold: First, the computational complexity of hyperbolic second-order operations (such as inverting the metric tensor or approximating the Riemannian Hessian) is typically $O(N^{2})$ or even $O(N^{3})$, while applying it to a subset reduces the complexity to $O(K^{2})$ or lower, significantly saving memory and computational power. Second, random sampling not only preserves the statistical characteristics of the overall momentum distribution (as the subset still represents the high-density regions), but also reduces noise accumulation, thus balancing efficiency, stability, and final convergence performance in large-scale parameter optimization. This step is typically employed for models with a large number of parameters, but can be selectively used for models with fewer parameters.

To capture the geometric relationships among modality-specific parameters, we project the model parameters from the conventional Euclidean space into a shared hyperbolic space. We adopt the Lorentz model as the representation of the hyperbolic space owing to its favorable mathematical properties that facilitate the definition of metrics and the computation of distances. The projection process transforms the original parameters into points that satisfy the constraints of the hyperbolic manifold, thereby laying a solid foundation for subsequent geometric computations. The Lorentz hyperbolic space is defined as follows:

(1) $$H^{n}=\left\{h\in R^{n+1}|{\langle h,h\rangle }_{c}=-1,h_{0}>0\right\},$$

where *h* denotes a point in the hyperbolic space, representing the coordinates of the parameter. $R^{n+1}$ indicates the (n+1)-dimensional space, containing both the time and space components. $h_{0}$ is the time component of the vector. ${\langle h,h\rangle }_{c}$ is the Lorentz inner product, which is required to be -1. $h_{0}>0$ is the time coordinate, which must be greater than zero. This equation introduces the definition of an (n+1)-dimensional Lorentz hyperbolic space $H^{n}$, where each point *h* must satisfy the constraint ${\langle h,h\rangle }_{c}=-1$. The equation represents a transformation into a Lorentz geometry, ensuring that the resulting vector *h* adheres to the corresponding transformation rules. The Lorentz inner product is defined as

(2) $${\langle h_{i},h_{j}\rangle }_{c}=-h_{i,0}h_{j,0}+\sum_{m=1}^{n}h_{i,m}h_{j,m},$$

where $h_{i}$ and $h_{j}$ denote two points in the hyperbolic space, representing the influence of the parameters. $h_{i,0},h_{j,0}$ are the time components of $h_{i}$ and $h_{j}$, respectively. $h_{i,m},h_{j,m}$ are the space–time components of $h_{i}$ and $h_{j}$ along the *m*-th dimension. $\sum_{m=1}^{n}$ is the summation over all space–time components. The Lorentz inner product represents the basic measure of the hyperbolic space, used to describe the relationship between two points in the curved space–time. The time components are negative, while the space components are positive, reflecting the characteristics of the space–time geometry. The projection formula is given by

(3) $$h_{k}=\pi_{H}\left(\theta_{k}\right)=\frac{\theta_{k}}{\sqrt{-{\langle \theta_{k},\theta_{k}\rangle }_{c}+\epsilon }},$$

where $h_{k}$ is the point in the curved space resulting from the projection of the parameter $\theta_{k}$. $\theta_{k}$ denotes the original model parameter, usually represented in terms of coordinate values (e.g., angle coordinates). ${\langle \theta_{k},\theta_{k}\rangle }_{c}$ is the Lorentz inner product used to normalize the transformation. ϵ is a small value (e.g., $10^{-6}$) used to ensure numerical stability during calculations.This formula maps the parameter $\theta_{k}$ obtained in the hyperbolic space to the curved space, producing the point $h_{k}$. The Lorentz inner product is used to normalize this transformation. The condition ${\langle h_{k},h_{k}\rangle }_{c}=-1$ is enforced, and ϵ is introduced to avoid computational errors when calculating the inverse of the norm. In practice, a parameter $\theta_{k}$ (such as a weight matrix in the network) is first flattened into a one-dimensional vector, and then projected using the aforementioned formula. The projected point $h_{k}$ lies on the hyperbolic manifold, providing a geometric representation for subsequent distance computations.

**Figure 3:**
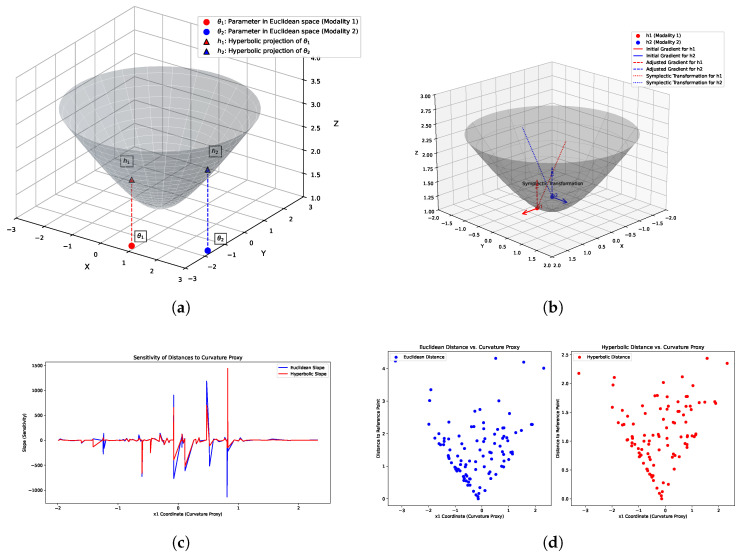
Visualization of the hyperbolic space diagram: (**a**) projection diagram of Euclidean space parameters into hyperbolic space, (**b**) symplectic transformation and gradient adjustment diagram, (**c**) slope plot of distance sensitivity to curvature proxy, and (**d**) scatter plot of distance versus curvature proxy.

In Figure 3a, we demonstrate how parameters from different modalities are mapped into hyperbolic space to better handle the geometric differences in multi-modal learning tasks. The figure shows the results of projecting two parameters from Euclidean space (parameters from modality 1 and modality 2) into hyperbolic space. In the 3D plot, red circles represent the parameters of modality 1, blue circles represent the parameters of modality 2, and black triangles indicate their projections in hyperbolic space. Through this mapping, the parameters are transformed from traditional Euclidean space into points in hyperbolic space, where the projection process effectively captures the curvature information of the manifold, thus making the features of the different modalities more coordinated. The dashed arrows in the figure represent the mapping path from the Euclidean space parameters to the hyperbolic space, illustrating the geometric transformation process. To demonstrate the advantages of hyperbolic cosine distance metrics on non-uniform curvature manifolds, we give two key visualizations (Figure 3c,d): a scatter plot illustrating the relationship between distance and curvature proxy and a slope plot reflecting the sensitivity of distance to curvature proxy. These plots are constructed by randomly sampling 100 points on a two-dimensional Lorentzian hyperbolic manifold. The first sampled point is designated as the reference, and both the Euclidean and hyperbolic distances from the reference point to all other points are computed. The scatter plot reveals that the Euclidean distance exhibits a relatively mild growth trend as the curvature proxy increases, whereas the hyperbolic distance demonstrates a much steeper growth in regions of high curvature, with the sampled points being more widely dispersed.

**Figure 4:**
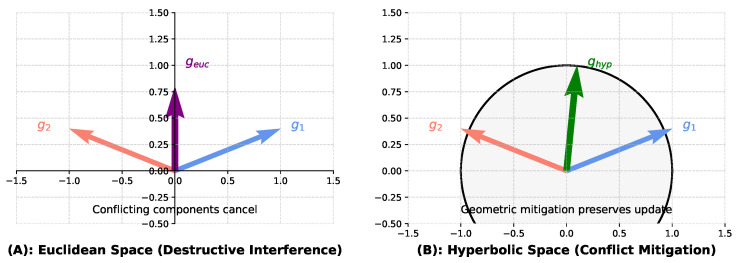
The diagram illustrates how hyperbolic projection mitigates gradient conflict. (**A**) In Euclidean Space: Two conflicting gradients, $g_{1}$ and $g_{2}$, partially cancel each other out (a ‘‘tug-of-war”), resulting in a small and inefficient update vector, $g_{euc}$. This represents destructive interference. (**B**) In Hyperbolic Space: The same gradients are projected onto the Poincaré disk. The hyperbolic geometry, leveraged by our optimizer, allows for a more effective aggregation. The resulting update, $g_{hyp}$, mapped back to the parameter space, avoids cancellation and produces a more decisive and stable update step.

To provide a clear intuition for why hyperbolic geometry is particularly well suited for resolving gradient conflicts, we present a conceptual diagram in Figure 4. This figure contrasts how conflicting gradients are aggregated in standard Euclidean space versus how they are handled via hyperbolic projection in our HC-SPA framework.

In the standard Euclidean setting (Figure 4A), if the gradients from the visible modality ($g_{1}$) and the infrared modality ($g_{2}$) point in opposing directions, their vector sum ($g_{euc}$) will be small. This is a classic ‘‘tug-of-war” scenario where training slows down or stalls because the conflicting information leads to destructive interference. In contrast, HC-SPA operates by projecting these gradients onto a hyperbolic manifold, visualized here as the Poincaré disk (Figure 4B). Due to the unique properties of this negatively curved space, the direct opposition between the vectors is geometrically reduced. Our optimization step, performed within this manifold, computes a resultant update that preserves the components of the gradients that are in agreement while minimizing their conflict. When this update is mapped back to the Euclidean parameter space for the model update, the resulting step ($g_{hyp}$) is more decisive and stable, leading to more efficient convergence and preventing the loss of information from either modality.

#### 3.2.2. Justification for Choosing the Lorentz Model

While several models exist for representing hyperbolic space, such as the popular Poincaré ball and Klein disk models, our selection of the Lorentz model was a deliberate choice motivated by its significant practical advantages for gradient-based optimization within modern deep learning frameworks.

First, fundamental geometric operations that are central to our algorithm have simpler and more direct closed-form expressions in the Lorentz model. These operations are primarily based on the Minkowski inner product, which is computationally efficient and straightforward to implement using standard tensor libraries. This contrasts with the Poincaré ball model, where corresponding operations like Möbius addition and gyrovectors are more complex.

Second, and critically for training deep neural networks, the Lorentz model offers superior numerical stability. In the Poincaré ball model, parameters approaching the boundary of the unit ball can cause their norms to approach infinity, leading to floating-point overflows and vanishing gradients, which destabilizes the optimization process. The Lorentz model, by embedding the space in a higher-dimensional pseudo-Euclidean space ($R^{n+1}$), elegantly sidesteps these boundary issues, providing a more robust and stable environment for large-scale, gradient-based optimization. Given these advantages in computational efficiency and numerical stability, we deemed the Lorentz model to be the most appropriate and reliable choice for our application.

#### 3.2.3. Computing the Hyperbolic Distance

After obtaining the hyperbolic space, we need to calculate the distance between the points in this space, which indicates the relative relationship between the parameters. To do this, we calculate the distance between different parameter points in the hyperbolic space. The hyperbolic distance is defined based on the position of the points in the hyperbolic space. Smaller parameters in the hyperbolic space should have a stronger influence, so the parameters need to be optimized to ensure their independence. The distance between the parameters should then be adjusted accordingly. The hyperbolic distance is defined as

(4) $$d\left(h_{i},h_{j}\right)=\mathrm{arcosh}\left(-{\langle h_{i},h_{j}\rangle }_{c}\right),$$

where $d(h_{i},h_{j})$ is the hyperbolic distance between $h_{i}$ and $h_{j}$. $h_{i},h_{j}$ are two points in the hyperbolic space whose distance is being calculated. ${\langle h_{i},h_{j}\rangle }_{c}$ are the Lorentz inner product between $h_{i}$ and $h_{j}$. arcosh(·) is the inverse hyperbolic cosine function, defined as $\mathrm{arcosh}\left(x\right)=\ln\left(x+\sqrt{x^{2}-1}\right)$, where x≥1. The hyperbolic distance is computed using the Lorentz inner product. The inverse hyperbolic cosine function (arcosh) is used to transform the distance. Since $h_{i}$ and $h_{j}$ satisfy the condition ${\langle h_{i},h_{j}\rangle }_{c}\geq 1$, the distance can be calculated. The distance value indicates the distance between the two points in the hyperbolic space. In the implementation, we first compute the Lorentz inner product $-{\langle h_{i},h_{j}\rangle }_{c}$ to obtain the distance between the points $h_{i}$ and $h_{j}$. Then, the arcosh function is applied to this result. The final distance calculation ensures that the relationship between the parameters is adjusted accordingly.

#### 3.2.4. Momentum Adjustment and Gradient Scaling

Traditional optimizers (e.g., SGD [34] with Momentum) update the momentum term based on the current gradient, but they do not consider the discrepancy in the magnitudes of the momentum terms between different models. The HC-SPA model introduces an additional auxiliary term that scales the momentum update, which ensures that the parameters remain in an optimal region of the solution space. This local coordination mechanism reduces momentum conflicts and facilitates smoother convergence to the optimal solution. The standard momentum update is given by

(5) $$m_{k}\leftarrow \beta m_{k-1}+\left(1-\beta \right)\nabla_{\theta_{k}}L,$$

where $m_{k}$ is the momentum at step *k*. β is the the momentum coefficient, typically set to 0.9, representing the historical momentum’s contribution. $\nabla_{\theta_{k}}L$ is the gradient of the loss function with respect to the parameters $\theta_{k}$. 1−β is the adjustment factor for the previous gradient. This formula is used to update the momentum term in momentum-based optimization methods, such as SGD with Momentum. It combines the previous momentum $m_{k-1}$ with the current gradient $\nabla_{\theta_{k}}L$, weighted by the factors β and 1−β. For HC-SPA, the update rule becomes

(6) $$m_{k}\leftarrow \beta m_{k-1}+\left(1-\beta \right)\nabla_{\theta_{k}}L+\alpha \sum_{j\neq k}w_{jk}\left(m_{j}-m_{k}\right),$$

where α is a coefficient for scaling the influence of the additional momentum term. $\sum_{j\neq k}$ is the sum over all parameters except for *k*, used to compute the interaction between the parameters. $w_{jk}$ represents the relationship between the parameters *j* and *k*. $m_{j},m_{k}$ is the momentum values for the parameters *j* and *k*, respectively. In the HC-SPA update rule, an auxiliary term is added to the standard momentum update, where the term $\sum_{j\neq k}w_{jk}\left(m_{j}-m_{k}\right)$ represents the interaction between the momentum at different parameter points. This term adjusts the momentum based on the relative differences between the parameters $m_{j}$ and $m_{k}$, ensuring smoother updates and reducing the conflict between different momentum components. The coupling weight $w_{jk}$ is defined as

(7) $$w_{jk}=\exp\left(-\gamma d\left(h_{k},h_{j}\right)\right),$$

where $w_{jk}$ is the coupling weight, which takes values in the range (0,1). γ denotes a scaling parameter that controls the sensitivity of the coupling weight to the distance. Typically set to 1.0. $d(h_{k},h_{j})$ is the distance between the parameters $h_{k}$ and $h_{j}$. exp(·) is the exponential function. The weight $w_{jk}$ is calculated using the exponential function, which takes the distance $d(h_{k},h_{j})$ between the two parameter points as input. The weight increases when the distance $d(h_{k},h_{j})$ is small, and it approaches 0 when the distance is large. The parameter update rule is $\theta_{k}\leftarrow \theta_{k}-\eta m_{k}$, where $\theta_{k}$ is the updated parameter, η is the learning rate that controls the step size for updating the parameters, and $m_{k}$ is the momentum term for the parameter $\theta_{k}$, which incorporates the gradient and coupling information. This rule is used to update the parameter $\theta_{k}$, where η is the learning rate, and $m_{k}$ is the momentum term for the parameter $\theta_{k}$. In the implementation, we first compute the momentum term $\beta m_{k}+\left(1↓\right)\nabla_{\theta_{k}}L$, and then update all parameters $\theta_{j}$. The coupling weight $w_{jk}$ is incorporated into the momentum term $m_{k}$, ensuring that the final update of $\theta_{k}$ incorporates information from other parameters and promotes convergence.

### 3.3. Synchronizing Gradient Update Phases

This subsection aims to create a symplectic structure by synchronizing the update phases of all parameters in tasks. To optimize the synchronization and stability of the system, we introduce gradient synchronization by using the method of gradient update. By performing synchronized updates across the system, we can improve the performance of the system and provide a better optimization direction for the parameters’ update phase.

#### 3.3.1. Gradient Synchronization

To achieve optimal synchronization and stability of the systems, we first define a new parameter phase and synchronize the updates across the different systems. By adjusting the synchronization direction and speed of the parameters, we can optimize the system performance, ensuring the stability and convergence of the model. For each parameter $\theta_{k}$, the gradient synchronization $p_{k}$ is defined as

(8) $$p_{k}=0,$$

where $p_{k}$ is the gradient synchronization term for the parameter $\theta_{k}$, initially set to zero. *k* is the index for the parameters in the system. 0 is the initial value for $p_{k}$, indicating the start of synchronization. In this equation, the gradient synchronization $p_{k}$ is initialized as zero, indicating the start of the parameter updates. The gradient updates and the synchronization speed are synchronized for stability. In the implementation, we first initialize the gradient synchronization $p_{k}$, and then apply the synchronization across all parameters. For example, in many systems, the parameter $\theta_{k}$ can represent RGB values. Once the synchronization is done, the system will use gradient synchronization to refine the parameter $\theta_{k}$, ensuring the stability and convergence of the system.

#### 3.3.2. Constructing the Update Rule

**Figure 5:**
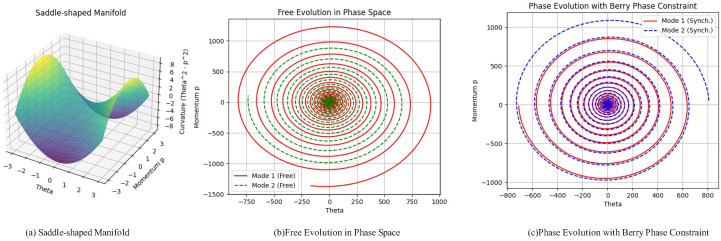
Comparison of free evolution and Berry phase constraint.

The momentum update rule is inspired by Hamiltonian mechanics. By optimizing the update process through the adjustment of parameters within the system’s configuration space, we can ensure stability in multi-dimensional tasks. This is achieved by optimizing the momentum parameters, ensuring a stable system that avoids erratic fluctuations, which are particularly crucial for the system’s convergence behavior. The momentum update formula is defined as

(9) $$p_{k}\leftarrow p_{k}-\eta \nabla_{\theta_{k}}L,$$

where $p_{k}$ is the momentum term for the parameter $\theta_{k}$. η is the learning rate, a scalar that controls the step size of the update. $\nabla_{\theta_{k}}L$ is the gradient of the loss function with respect to the parameter $\theta_{k}$. The momentum update $p_{k}$ is adjusted based on the parameter $\theta_{k}$. The formula resembles the acceleration of particles in a physical system, where the learning rate η influences the magnitude of the update. Then parameter update

(10) $$\theta_{k}\leftarrow \theta_{k}+\eta M_{k}^{-1}p_{k},$$

where $\theta_{k}$ is the parameter to be updated, both before and after the update. $M_{k}$ is the quality matrix, which is positive definite and typically controls the curvature of the parameter space. $M_{k}^{-1}$ is the inverse of the quality matrix, used to adjust the momentum. In this update rule, the momentum $p_{k}$ is adjusted, and then it is used to update the parameter $\theta_{k}$. The inverse of the quality matrix $M_{k}$ influences the size of the parameter update, ensuring a more refined and efficient update direction. In practice, the matrix $M_{k}$ can be simplified as the identity matrix *I*, making the update rule $\theta_{k}\leftarrow \theta_{k}+\eta p_{k}$. This simplification allows for faster computation, as the Euler method ensures stable updates. The specific implementation involves computing the momentum $p_{k}$ and then updating the parameter $\theta_{k}$, ensuring convergence by controlling the momentum’s magnitude at each step. Figure 5 illustrates the evolution of the system’s phase space trajectory under both free evolution and imposed synchronization constraints. This figure visually demonstrates the impact of phase synchronization mechanisms on the system’s evolution and validates the effectiveness of synchronization mechanisms in enhancing system stability and cooperation. Through this synchronization constraint, the system’s phase difference converges, highlighting how synchronization constraints can effectively optimize the system’s cooperative evolution.

Our method avoids this by introducing what is known in Hamiltonian mechanics as a symplectic structure. An intuitive analogy is to think of it as a synchronization rule for the parameter updates. The path labeled “Berry phase constraint” represents the optimization trajectory guided by HC-SPA. In this context, the “constraint” is our phase alignment mechanism. It forces the gradient updates into a synchronized rhythm, eliminating the destructive interference. This enforcement of a shared phase results in a smooth, stable, and more direct convergence path, which is why our method is more effective at preserving details and producing a high-quality, artifact-free fused image.

#### 3.3.3. Phase Coordination Mechanism

To further improve the coordination efficiency in multi-tasking, we use a phase coordination method, which optimizes the parameter update steps by calculating the adjustment of the distances between parameters. Through this phase coordination, the system can maintain a balance among multiple parameters, enhancing the overall coordination effect during the model training process, as follows:

(11) $$p_{k}\leftarrow p_{k}-\eta \nabla_{\theta_{k}}L+\beta \sum_{j\neq k}w_{jk}\left(p_{j}-p_{k}\right),$$

where $p_{k}$ is the update for the *k*-th parameter (on the left side is the current update; on the right side is the updated value). η is the learning rate, controlling the step size for each update. $\nabla_{\theta_{k}}L$ is the gradient of the loss function with respect to the *k*-th parameter. β is the coefficient for the phase coordination term, controlling its strength. $w_{jk}$ is the coupling weight between the *j*-th and *k*-th parameters, representing the relationship strength between their updates. This formula introduces a phase coordination term $\sum_{j\neq k}w_{jk}\left(p_{j}-p_{k}\right)$ in the update step. With this term, the parameter updates are influenced by other related parameters, helping to achieve coordination between different parameters during the optimization process, ensuring the stability and consistency of the model throughout the training process. The coupling weight $w_{jk}$ is defined as

(12) $$w_{jk}=\exp\left(-\gamma d\left(h_{k},h_{j}\right)\right),$$

where $w_{jk}$ is the coupling weight, which takes values in the range (0,1). γ denotes a scaling factor that controls the strength of the coupling effect, typically set to 1.0. $d(h_{k},h_{j})$ indicates the distance between the parameters $h_{k}$ and $h_{j}$, where smaller distances lead to stronger coupling and larger distances lead to weaker coupling. $h_{k}$ and $h_{j}$ are the parameters, whose relationship is represented by the coupling weight. exp(·) is the exponential function. The coupling weight $w_{jk}$ is defined by a function that scales the distance $d(h_{k},h_{j})$ between parameters. As the distance $d(h_{k},h_{j})$ decreases, the coupling weight $w_{jk}$ approaches 1, enhancing the coupling effect. When the distance increases, $w_{jk}$ approaches 0, reducing the coupling effect. In the phase coordination mechanism, we compute the distance $d(h_{k},h_{j})$ and use it to adjust the coupling weight. During each optimization iteration, we calculate the coupling term for all parameters. This approach ensures that parameters with similar values or locations in the space will synchronize their updates, promoting convergence in the optimization process. Figure 3b provides a conceptual illustration of this symplectic update process. The gray funnel-shaped surface in the figure should not be interpreted as the loss landscape itself, but rather as a conceptual iso-energy surface derived from our Hamiltonian framework. All points on this surface represent states with an identical Hamiltonian value (*H*). The initial gradients from two modalities (solid red and blue arrows) are first mapped to this surface as momentum vectors. The symplectic transformation then performs a phase rotation on these vectors along the surface. This produces the adjusted gradients (dashed arrows). Crucially, this adjustment modifies the direction (phase) of the gradients to bring them into alignment, while preserving their magnitude (energy), as they remain on the same iso-energy surface. This mechanism of directional alignment without altering gradient magnitude is the key to how HC-SPA resolves conflicts and synchronizes updates.

### 3.4. Integration with Deep Learning Models and Modularity

A key advantage of the HC-SPA framework is its design as a modular, plug-and-play optimizer, allowing for straightforward integration with various deep learning architectures without altering their underlying structure. To clarify how HC-SPA is applied, we detail the integration process here.

Our method functions as a direct replacement for standard optimizers such as SGD or Adam. The entire novelty of HC-SPA is encapsulated within the optimizer’s “step()” function. The training process for a given fusion model, like SeaFusion, follows the following standard procedure: 1. A forward pass is performed to generate the fused output. 2. The loss function is computed. 3. The standard “loss.backward()” operation is called. This uses the deep learning framework’s automatic differentiation engine to compute the gradients “$\nabla_{\theta }L$” for all trainable parameters “θ” of the model. Our method does not modify this backpropagation step. 4. The “optimizer.step()” function is called. At this stage, HC-SPA takes the pre-computed gradients and applies its unique update rule. It internally groups the parameters by modality, calculates the hyperbolic coupling weights “$w_{jk}$”, and executes the symplectic phase alignment update as described in Equation (11) to update the model’s weights.

Therefore, HC-SPA is applied not to specific layers but to the entire set of trainable model parameters passed to it during initialization. The optimizer itself is responsible for logically grouping these parameters to manage their interactions. This modularity ensures that HC-SPA is highly transferable and can be readily applied to other multimodal fusion tasks.

## 4. Experiments

This subsection first presents the experimental configuration and implementation details. To validate the effectiveness and robustness of the hyperbolic cosine-based Shinn–Sao phase alignment (HC-SPA) framework in enhancing multimodal fusion tasks, we design and conduct three comprehensive experiments focusing on integrating HC-SPA into two different fusion models, SeAFusion [10] and SDCFusion [35], as well as demonstrating the effectiveness across different datasets. The first experiment applies HC-SPA to the SeAFusion model to evaluate its impact on optimizing stability and improving segmentation performance in RGB and infrared image fusion. The second experiment incorporates HC-SPA into the SDCFusion model to verify whether HC-SPA also yields favorable optimization results across different architectures. In the third experiment, we explore the impact of adjustments under different optimization parameters on the model, and further analyze the actual effect of the parameter layer.

### 4.1. Experimental Configurations

We conduct comprehensive experiments across four datasets, namely, MSRS [36], RoadScene [37], and TNO [38], to thoroughly evaluate the performance of our models in multimodal image fusion tasks. The models evaluated include SeAFusion and SDCFusion, both integrated with the HC-SPA optimizer to enhance RGB and infrared fusion. SeAFusion emphasizes feature fusion for semantic segmentation, while SDCFusion focuses on achieving spatiotemporal consistency in multimodal fusion. We also compared HC-SPA with other optimizers such as SGD [34], RMSProp [39], Adadelta [40], and AMSGrad [41] to demonstrate its advantages in gradient coordination and fusion performance.

To objectively assess fusion quality, multiple evaluation metrics are employed. The Mean Squared Error (MSE) [42] measures pixel-wise differences between the fused and reference images, with lower values indicating better accuracy. The Standard Deviation (SD) [43] reflects image contrast, with higher values indicating stronger contrast. Peak Signal-to-Noise Ratio (PSNR) [44] assesses the overall image fidelity, with higher values indicating less distortion. Mutual Information (MI) [45] quantifies the shared information between input images (RGB and infrared) and the fused result, reflecting the fidelity of fusion. Visual Information Fidelity (VIF) [46] evaluates perceptual quality by measuring how well the fused image preserves visual features. Average Gradient (AG) [47] assesses the richness of textures and edge clarity, with higher values indicating more detailed images. The Correlation Coefficient (CC) [48] measures the structural and content similarity between fused and input images. Entropy (EN) quantifies the information content, where lower entropy indicates clearer fusion results. Normalized Absolute Bias Function (Nabf) [49] evaluates the overall error of the fused image, with lower values denoting higher accuracy. The Structural Similarity Index (SSIM) [50] measures the structural similarity between the fused and reference images, with higher values indicating better preservation of structural information.

### 4.2. Implementation Details

The fusion model is trained on the MSRS, RoadScene, and TNO datasets. Specifically, on the MSRS dataset, the training consists of 10 epochs with a batch size of 4 and a single iteration per epoch (compared with 4 iterations in the original paper). The initial learning rate is set to 0.01, with a decay factor of 0.75 applied at each epoch to ensure stable convergence. The HC-SPA optimizer is employed, configured with hyperparameters: learning rate (lr) = 0.01, beta = 0.9, alpha = 2, gamma = 5.0, and epsilon = 1 × 10^−6^. (Beta is the momentum coefficient, which controls the exponential moving average of gradients. It balances the influence between past and current gradients to smooth and accelerate the optimization process. alpha is the coupling strength parameter, which adjusts the degree to which hyperbolic momentum interactions influence the optimization process. It determines the extent to which global momentum information contributes to parameter updates. Gamma is the distance sensitivity parameter, which affects the decay rate of the coupling weights as the hyperbolic distance between momenta increases. It defines the geometric range of momentum interactions.) In the SDCFusion experiments, we also use the MSRS, RoadScene, and TNO datasets, training for 200 epochs, with a batch size of 4. The initial learning rate is set to 0.001 and is decayed polynomially with an exponent of 0.9. A warm-up phase of 1000 iterations (starting from 1 × 10^−5^) is implemented. Due to the large model size, a subset of sampling parameters is used to reduce computational costs, with a current sampling size of 1000. The HC-SPA optimizer’s hyperparameters are configured as follows: lr = 0.001, beta = 0.9, alpha = 1.5, gamma = 10.0, epsilon = 1 × 10^−6^, and a weight decay of 5 × 10^−4^ to regularize parameter updates. All experiments are conducted on NVIDIA 3090 Ti GPUs.

### 4.3. Comparative Experiments

To thoroughly evaluate the effectiveness of the proposed method in enhancing fusion performance, we first use the optimization approach to SeAFusion. Subsequently, we conduct comparative experiments with five other methods on the aforementioned publicly available datasets.

**Figure 6:**
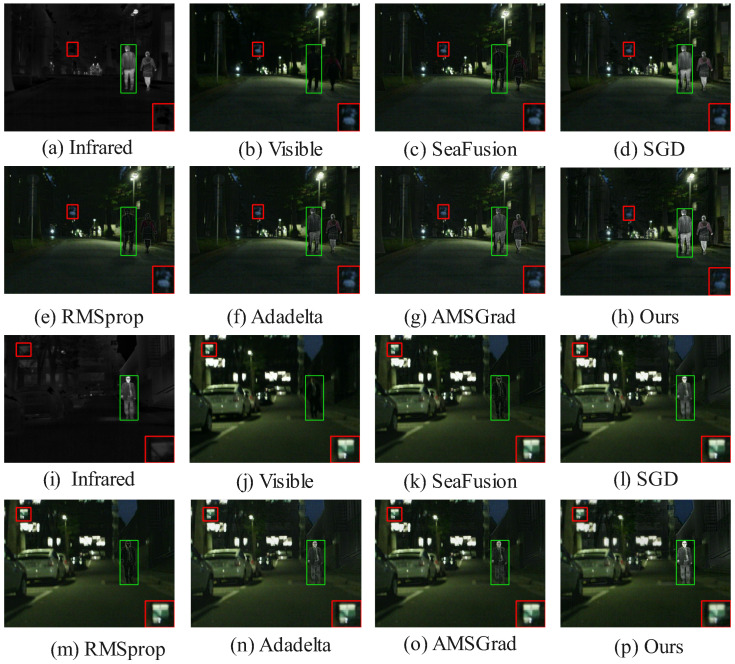
Comparison of fusion results using different methods in the MSRS dataset (nighttime).

**Figure 7:**
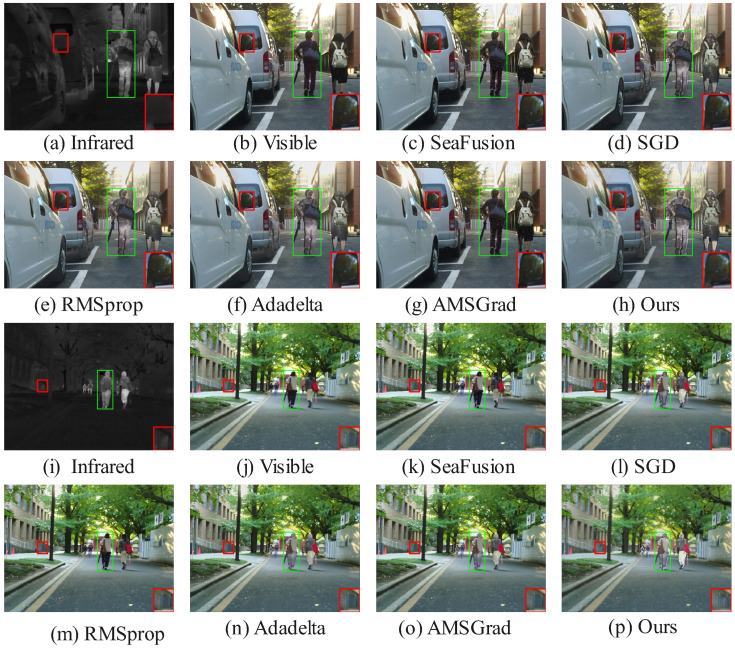
Comparison of fusion results using different methods in the MSRS dataset (daytime).

Figure 6 and Figure 7 showcase qualitative results from the MSRS dataset, where HC-SPA outperforms methods like SeaFusion, SGD, RMSProp, Adadelta, and AMSGrad in both night- and daytime scenes. In night scenes, SeaFusion maintains brightness but blurs textures, with faint light points and flat contrast in the red-boxed region, while pedestrian edges in the green-boxed region diffuse grayly, lacking details like clothing wrinkles. SGD boosts brightness but adds noise, distorting the image; RMSProp and Adadelta reduce noise but flatten details; and AMSGrad balances brightness and shape yet leaves pedestrian contours blurred. HC-SPA, however, restores sharp light point shapes and pedestrian outlines with vivid wrinkles, excelling in low-light detail and contrast. In daytime scenes, SeaFusion reduces noise but obscures pedestrian edges and background details, SGD enhances contrast but introduces noise, RMSProp and Adadelta lose reflective details, and AMSGrad blurs railings and textures. HC-SPA shines with clear car window contours, natural halo effects, and rich clothing details, ensuring high contrast and discernibility in multimodal fusion.

**Figure 8:**
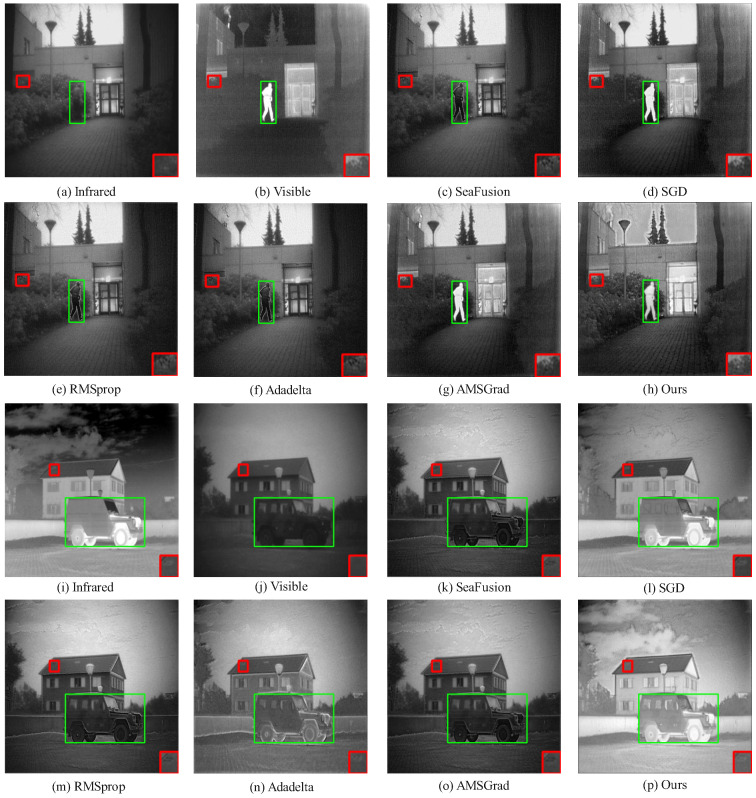
Comparison of fusion results using different methods in the TNO dataset.

**Figure 9:**
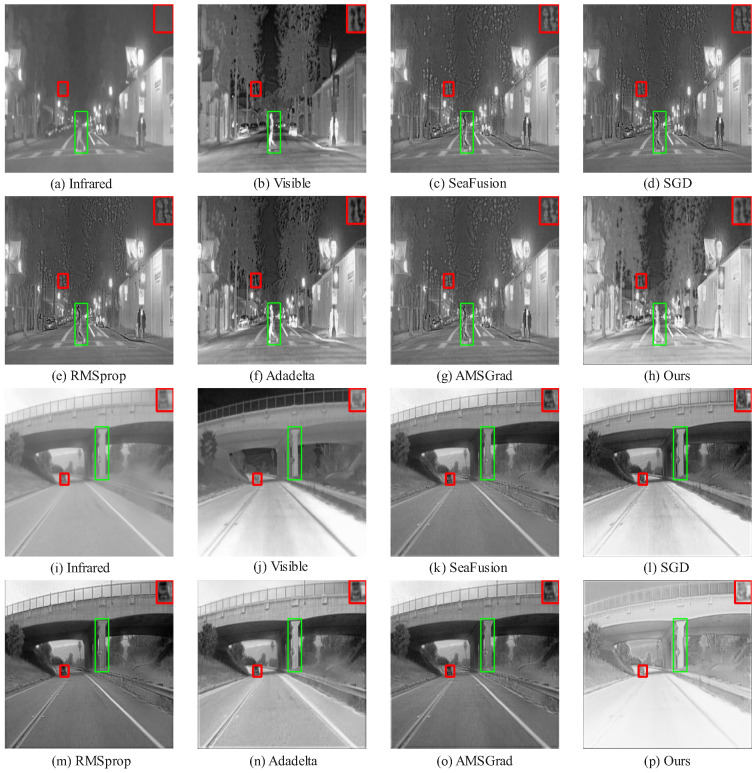
Comparison of fusion results using different methods in the RoadScene dataset.

On the TNO and RoadScene datasets (Figure 8 and Figure 9), HC-SPA surpasses conventional optimizers (SGD, RMSProp, Adadelta, AMSGrad) and baseline fusion models by preserving salient objects with minimal interference. In green-boxed salient areas, RMSProp and Adadelta integrate thermal data but add noise and blurring backgrounds, while HC-SPA preserves natural visible textures with little disruption. In red-boxed edge regions, SGD and AMSGrad lose fine boundary details, and SeaFusion introduces thermal artifacts, but HC-SPA maintains sharp contours, blending thermal cues and visible richness effectively, matching original infrared pixel distributions. Even in poor fusion scenarios, where only Adadelta competes in balancing visible and thermal data, HC-SPA excels in detail fidelity, low-light adaptability, and contrast enhancement, proving its robustness in complex multimodal tasks.

#### Quantitative Results

**Figure 10:**
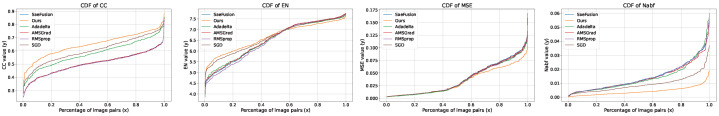
Comparative plots of the SeaFusion model employing different optimizers in the MSRS dataset across the CC, EN, MSE, and Nabf metrics.

The quantitative experiments are conducted on the MRSR dataset (Table 1 and Figure 10), and the proposed optimizer (Ours) demonstrates significant advantages in both image quality and fusion performance. Specifically, Ours achieves the lowest MSE of 0.034, representing an approximately 8.1% reduction compared with the second-best method, RMSprop (0.037), indicating superior pixel-level reconstruction accuracy. Meanwhile, it attains the highest SD of 41.46, reflecting a richer pixel distribution and more comprehensive detail restoration. In terms of PSNR, Ours reaches 64.49 dB, outperforming SGD (64.03 dB) by 0.46 dB, further validating its excellence in balancing denoising and fidelity. The AG, a key indicator for image enhancement, peaks at 3.286, approximately 6.8% higher than SeaFusion, indicating more effectively enhanced edges and textures in the fused images.

Additionally, Ours achieves near or superior performance on structural fidelity metrics, with a CC of 0.674 and a SSIM of 0.4781. Combined with its exceptionally low noise metric Nabf (only 0.003), the results demonstrate an optimal trade-off between global structural preservation and noise suppression.

**Figure 11:**
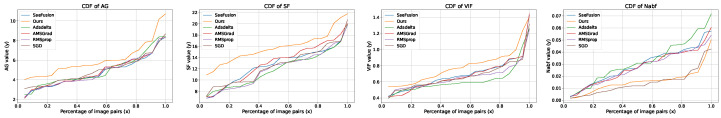
Comparative plots of the SeaFusion model employing different optimizers in the TNO dataset across the AG, SF, VIF, and Nabf metrics.

**Figure 12:**
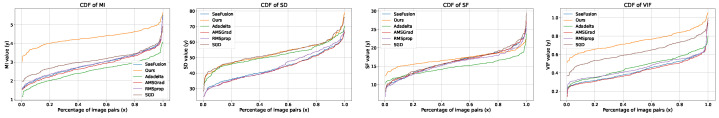
Comparative plots of the SeaFusion model employing different optimizers in the RoadScene dataset across the MI, SD, SF, and VIF metrics.

Cross-domain evaluations in the TNO (Table 2 and Figure 11) and RoadScene (Table 3 Figure 12) datasets further highlight the generalization capability and robustness of the proposed optimizer. In the TNO dataset, Ours records an MSE of 0.019, slightly higher than RMSprop (0.016), but surpasses all baselines in terms of Visual Information Fidelity (VIF = 0.878) and Average Gradient (AG = 5.993). The VIF improves by approximately 16.3% over the next-best SGD (0.756), while AG outperforms Adadelta (4.243) by nearly 41.2%. Furthermore, it achieves a Correlation Coefficient (CC) of 0.624 and a spatial frequency (SF) of 12.96, indicating superior complementarity in cross-spectral information fusion and finer structural depiction, with the noise metric Nabf remaining at a low level of 0.012.

In the more challenging RoadScene thermal infrared dataset, Ours continues to deliver comprehensive improvements: MSE decreases to 0.048, outperforming the best baseline SGD (0.051); SD increases to 47.40, a 12.3% gain over Adadelta (42.18); and VIF and entropy (EN) rise to 0.847 and 7.405, respectively, while CC reaches 0.284 and SF hits 15.90, both surpassing all existing baselines. Overall, these cross-dataset comparisons not only verify the proposed optimizer’s consistent enhancement of fusion quality under varying conditions but also emphasize its superior capacity in adaptive learning of multimodal features and noise suppression.

### 4.4. Analysis of Metric Variations and Optimizer Efficacy

The qualitative advantages showcased in Figure 6 through Figure 9 are not just perceptual; they are direct manifestations of the optimization dynamics fostered by HC-SPA, which are in turn captured by the quantitative metrics. A closer examination reveals this strong link between visual evidence and metric performance, as follows:

Detail and Contrast (SD and AG): The superior Standard Deviation (SD) and Average Gradient (AG) values reported in Table 1 are the numerical representation of enhanced detail and contrast. Theoretically, our hyperbolic gradient coordination prevents the high-frequency texture information (e.g., from the visible modality) from being suppressed by the strong, low-frequency thermal signatures (from the infrared modality). This is visually corroborated in the nighttime scene in Figure 6. The sharp, vivid wrinkles on the pedestrian’s clothing (green box) and the distinct halo around the light source (red box) in our result directly contribute to a higher AG and SD. In contrast, the blurred or flattened results from other optimizers reflect their struggle to balance these conflicting gradient signals, leading to lower scores.

Fidelity and Information Synergy (PSNR, CC & VIF): The higher Peak Signal-to-Noise Ratio (PSNR), Correlation Coefficient (CC), and Visual Information Fidelity (VIF) scores indicate that HC-SPA produces a fused image that is a more faithful and synergistic combination of the source inputs. This is achieved by the symplectic phase alignment, which ensures stable, synchronized updates and minimizes the generation of artifacts. For example, in the daytime scene in Figure 7, the clear car window contours and well-defined background railings (red box) in our fused image demonstrate high structural fidelity, which is reflected in a high CC and SSIM. Similarly, in Figure 8, our method’s ability to preserve the natural texture of the background from the visible image while cleanly integrating the salient person from the infrared image (green box) leads to a higher VIF, as it better matches human visual perception. Methods that introduce noise (like RMSprop) or artifacts naturally score lower on these fidelity metrics.

Thus, the significant variations in these key metrics are not arbitrary; they provide quantitative validation for the qualitative improvements observed. They confirm that HC-SPA’s geometric approach to optimization directly translates to more detailed, stable, and informative fusion outcomes.

### 4.5. Semantic Segmentation Performance

**Figure 13:**
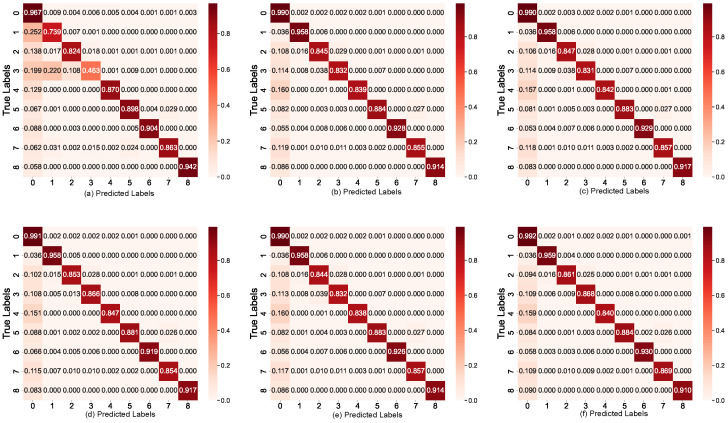
Comparison of segmentation results using different methods on the MSRS dataset: (**a**) Adadelta, (**b**) AMSGrad, (**c**) RMSprop, (**d**) SGD, (**e**) SeaFusion, and (**f**) Ours.

**Figure 14:**
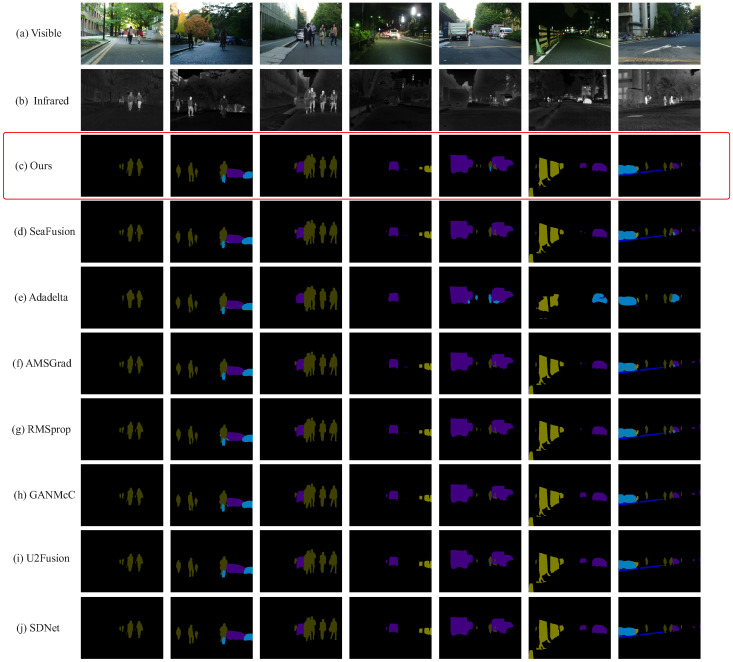
Comparison of segmentation results on the MSRS dataset using various models and optimization methods. The first row shows light images, while the second row displays infrared images. The proposed outperforms other models, including SeaFusion (with AMSGrad, RMSprop, and Adadelta optimizers), GANMcC, U2Fusion, and SDNet, especially in handling complex backgrounds and fine details. Ours achieves superior segmentation accuracy and detail preservation, particularly in infrared images, highlighting its effectiveness in challenging scenarios. The content in the red box is our result display.

To further assess the quality of the fusion results, we conduct segmentation experiments on the fused images (Table 4 and Figure 13), comparing Ours with state-of-the-art models such as GANMcC [51], U2Fusion [52], and SDNet [53], as well as SeaFusion combined with optimizers, including AMSGrad, RMSProp, Adadelta, Our optimizer. The proposed method demonstrates a clear advantage in segmentation tasks under complex backgrounds. As shown in Figure 14, Ours achieves higher accuracy and better detail preservation in both visible and infrared image segmentation. In particular, it maintains sharp object contours and high precision under low-light conditions. Although SeaFusion generates reasonable results, its edge precision is limited, often leading to segmentation overlaps or omissions. GANMcC, U2Fusion, and SDNet also perform less favorably on infrared images, frequently exhibiting recognition errors and incomplete segmentation. Overall, Ours outperforms other methods in terms of accuracy and fine-grained segmentation, especially in multi-object complex scenes.

### 4.6. Validation Experiment

To validate the effectiveness of the proposed method in enhancing fusion performance, we applied this optimizer to the recently developed multimodal fusion model, SDCFusion. Experiments are conducted using two distinct sets of optimizer hyperparameters and three publicly available datasets. The results demonstrate that our method outperforms the original model across both hyperparameter settings.

#### 4.6.1. Qualitative Results

**Figure 15:**
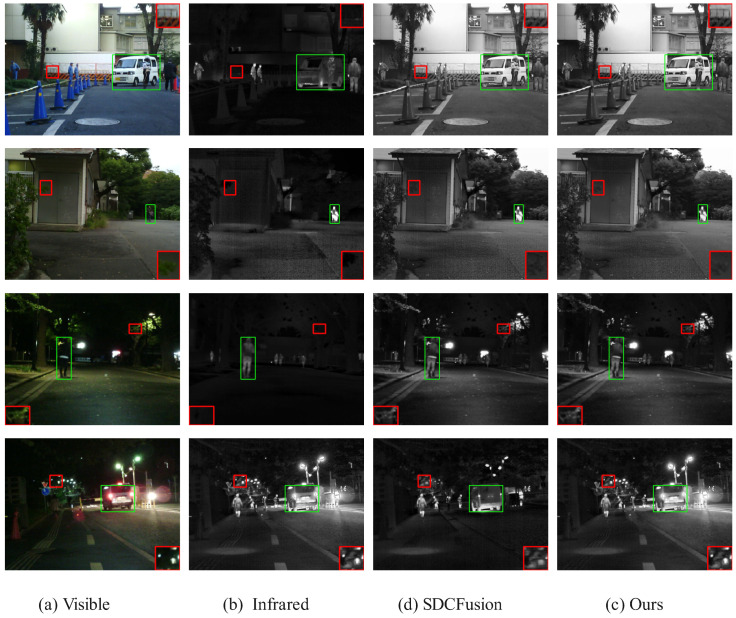
Comparison of fusion results in the MSRS dataset. The red box shows an enlarged detail of the image content, and the green box shows the prominent targets in the image. If there is no special explanation in the subsequent figure, it is synonymous with this.

**Figure 16:**
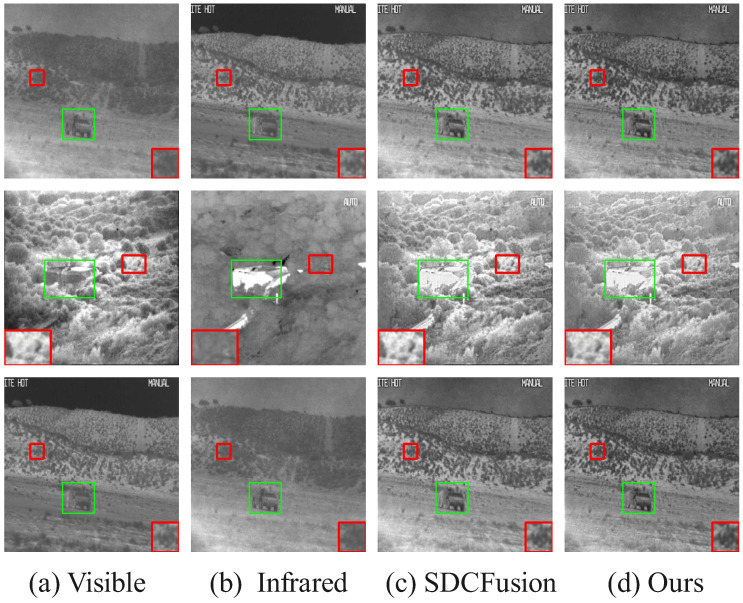
Comparison of fusion results of tested solutions in the RoadScene dataset. The red box shows an enlarged detail of the image content, and the green box shows the prominent targets in the image.

**Figure 17:**
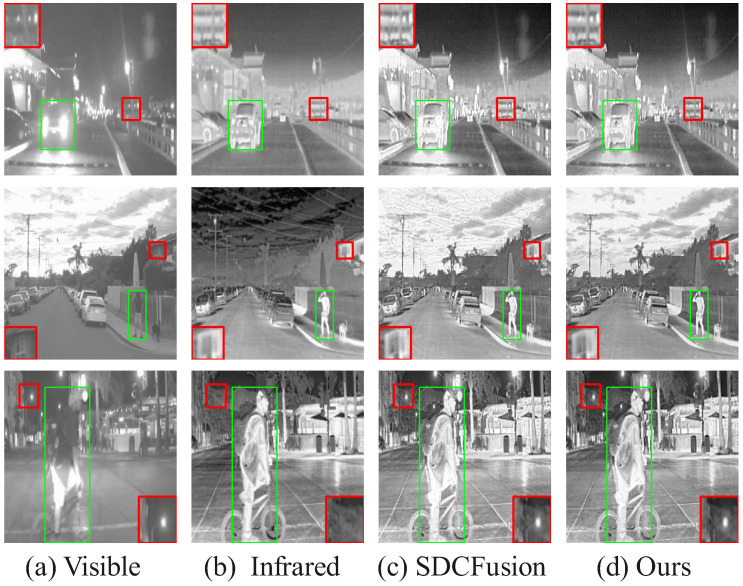
Comparison of fusion results of tested solutions in the TNO Dataset. The red box shows an enlarged detail of the image content, and the green box shows the prominent targets in the image.

Figure 15, Figure 16 and Figure 17 presents a comparison of the fusion results of our model in the different datasets. Each column in the figure represents different image modalities and fusion methods: (b) visible shows the original visible image of a daytime scene; (a) infrared provides the infrared image under low-light conditions, highlighting the thermal imaging information; (c) SDCFusion is the baseline fusion method, which, although achieving relatively good fusion results, lacks detail preservation, especially under low-light conditions; (d) our model incorporates the HC-SPA optimizer, making the salient objects more prominent while improving detail preservation with greater precision. Compared with SDCFusion, our model enhances the visible information under bright conditions and effectively supplements the infrared details under low-light conditions, offering a clearer overall performance. After only minimal training, our model achieves satisfactory results, fully demonstrating the effectiveness of the HC-SPA optimizer in optimizing model performance.

#### 4.6.2. Quantitative Results

Table 5 presents the quantitative evaluation results of SDCFusion, Ours1, and Ours2 across the TNO, RoadScene, and MSRS datasets, providing a systematic comparison of their image fusion capabilities. Ours1 and Ours2 are derived by applying the HC-SPA optimizer to the SDCFusion model, with Ours1 trained for 100 epochs and both Ours2 and the baseline SDCFusion trained for 200 epochs. In the TNO dataset, Ours1 and Ours2 outperform SDCFusion in key metrics, achieving higher Peak Signal-to-Noise Ratio (PSNR) values of 61.99 and 62.03, respectively, compared with 61.48 for SDCFusion, indicating improved image quality. The mean squared error (MSE) decreased to 0.041 (Ours1) and 0.040 (Ours2) from 0.046 (SDCFusion), reflecting reduced prediction errors, while mutual information (MI) increased to 2.387 (Ours1) and 2.365 (Ours2) from 2.236 (SDCFusion), demonstrating enhanced information preservation. In the RoadScene dataset, Standard Deviation (SD) rose significantly to 47.87 (Ours1) and 50.43 (Ours2) from 44.47 (SDCFusion), and MI improved to 3.340 (Ours1) and 3.187 (Ours2) from 2.620, alongside a reduction in MSE to 0.081 and 0.085 from 0.088, highlighting greater contrast and accuracy. Similar improvements are observed in the MSRS dataset. However, a minor reduction in spatial consistency deviation (SCD) is noted across all datasets, with values of 1.836 (Ours1) and 1.850 (Ours2) compared with 1.866 (SDCFusion) in TNO, and 1.647 (Ours1) and 1.620 (Ours2) compared with 1.778 (SDCFusion) in RoadScene, suggesting a slight trade-off in spatial consistency. These results collectively validate the efficacy of the HC-SPA optimizer in enhancing image fusion quality and preserving informative content, while providing insights for future model optimization.

### 4.7. Hyperparameter Experiment

**Figure 18:**
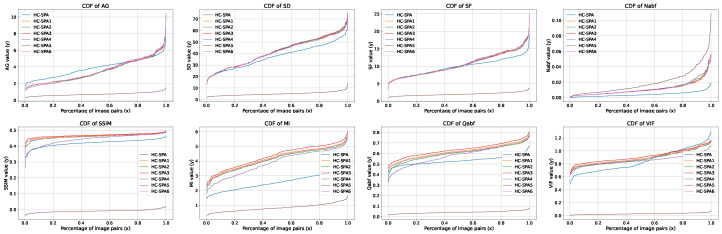
Comparative plots across different fusion quality metrics: AG, SD, SF, Nabf, SSIM, MI, Qabf, and VIF.

To compare the experimental results under different parameters, we present an analysis of the hyperparameter experiments conducted with the HC-SPA optimizer integrated into the SeaFusion model, utilizing the MSRS dataset. Table 6 and Figure 18, present the results of hyperparameter experiments evaluating seven configurations of the HC-SPA optimizer integrated into the SeaFusion model in the MSRS dataset, focusing on the effects of varying alpha, beta, and gamma on key metrics such as Mean Squared Error (MSE), Peak Signal-to-Noise Ratio (PSNR), and Visual Information Fidelity (VIF). The baseline configuration (alpha = 2.0, beta = 0.90, gamma = 5.0) achieved optimal performance, recording the lowest MSE (0.034) and highest PSNR (64.49), indicating superior image quality and minimal error. However, it is surpassed in VIF (0.843) and spatial frequency (SF, 8.769) by other configurations, suggesting trade-offs in visual fidelity and texture detail. These findings emphasize the importance of carefully tuning the interaction between alpha and gamma to preserve optimization stability, particularly in larger models, while beta contributes modest improvements to stability through enhanced smoothing dynamics.

A closer examination of the results in Table 6 further clarifies the model’s robustness to these hyperparameter variations. **Sensitivity to α, β, and γ:** The model demonstrates varied sensitivity to each hyperparameter. For instance, when comparing HC-SPA2 (α=1.0) and HC-SPA5 (α=2.0), where other parameters are held constant, a significant drop in PSNR from 64.08 to 49.83 is observed, indicating that the method is sensitive to a larger α and that a value of 1.0 is a more stable choice. Conversely, the model is highly robust to changes in β. The performance metrics for HC-SPA2 (β=0.90) and HC-SPA3 (β=0.95) are nearly identical, confirming that the choice of β is not a critical factor within this range. Most importantly, addressing the stability with respect to γ, we observe only graceful degradation across a wide spectrum. As γ increases from 2.0 (HC-SPA1) to 10.0 (HC-SPA2) and finally to 20.0 (HC-SPA4), the PSNR remains consistently high (63.84, 64.08, and 63.75 respectively), without any sharp decline. This confirms that our method is not “brittle” and maintains high performance across a broad range of γ values.

Furthermore, our choice to use full parameter updates instead of sampled subsets is a deliberate theoretical decision for optimization on the Lorentz manifold. While sampling can be effective in Euclidean space, the geometric integrity of the Lorentz manifold is paramount. Using the full parameter set ensures that the computed gradient is a true vector in the tangent space, guaranteeing that the update follows a valid geodesic path. A sampled subset would introduce stochastic noise that could easily corrupt the gradient direction, causing the update to “fall off” the manifold, which would violate its geometric constraints and lead to severe training instability. Therefore, the full update strategy is a necessary choice to ensure the theoretical soundness and convergence stability of our approach. However, if the model parameters are too large, using full parameter updates will affect efficiency and value, and sampling parameter updates can still be attempted.

## 5. Conclusions

In this paper, we proposed the Hyperbolic Cosine-Based Hamiltonian Phase Alignment fusion optimizer (HC-SPA), a novel approach designed to directly address the gradient conflicts and phase mismatches inherent in multimodal image fusion. Our experimental results on multiple benchmark datasets robustly demonstrate that by operating in a geometric space tailored for optimization, HC-SPA significantly enhances fusion quality, particularly in challenging low-light conditions. It produces images with more prominent salient objects and refined details. This confirms the practical implication that addressing the underlying optimization dynamics offers a powerful alternative to purely architectural innovations for advancing fusion performance.

Despite its promising performance, HC-SPA has limitations that warrant discussion. The primary trade-off is between performance and ease of use, stemming from its sensitivity to key hyperparameters. As analyzed in our hyperparameter experiments, the choice of these parameters is critical, and suboptimal settings can impact the fusion outcome. This indicates that while the optimizer is powerful, its application to new tasks requires a careful tuning process. Additionally, the symplectic transformations introduce a modest computational overhead compared with standard optimizers, which could be a consideration for real-time applications on resource-constrained devices.

Future work can proceed in several promising directions to address these limitations and expand upon our findings. First, developing an adaptive mechanism to automatically tune the key hyperparameters would greatly enhance the optimizer’s usability and robustness. Second, improving computational efficiency, perhaps by exploring approximations of the symplectic transformations, would broaden its applicability. For example, extending the application of HC-SPA beyond image fusion to other multi-modal domains, such as vision language models or medical imaging analysis, could validate the generalizability of our gradient synchronization approach to a wider range of machine learning challenges.

## Figures and Tables

**Table 1:** A comparison of the performances of different detection models on various metrics in the MRSR dataset.

Model	MSE	SD	PSNR	AG	CC	EN	Nabf	SSIM
SeaFusion	0.039	40.20	63.89	3.077	0.540	6.659	0.014	0.3104
SGD	0.038	40.13	64.03	3.040	0.611	6.703	0.008	0.4951
RMSprop	0.037	39.37	64.06	3.036	0.536	6.607	0.013	0.3142
Adadelta	0.039	39.46	63.80	3.015	0.596	6.643	0.014	0.3104
AMSGrad	0.038	40.02	63.96	3.096	0.542	6.658	0.013	0.3026
Ours	0.034	41.46	64.49	3.286	0.674	6.675	0.003	0.4781

**Table 2:** A comparison of the performances of different detection models on various metrics in the TNO dataset.

Model	MSE	SD	VIF	AG	CC	SF	Nabf	SSIM
SeaFusion	0.024	41.23	0.676	3.879	0.339	11.17	0.043	0.1489
Adadelta	0.024	39.87	0.589	4.243	0.535	8.797	0.036	0.3802
AMSGrad	0.024	41.53	0.648	3.886	0.400	10.61	0.041	0.1272
RMSprop	0.016	48.80	0.678	3.823	0.399	8.746	0.040	0.1759
SGD	0.086	37.69	0.756	4.115	0.611	9.644	0.017	0.4227
Ours	0.019	41.66	0.878	5.993	0.624	12.96	0.012	0.4038

**Table 3:** A comparison of the performances of different detection models on various metrics in the RoadScene dataset.

Model	MSE	SD	VIF	EN	CC	SF
SeaFusion	0.062	38.66	0.483	6.885	0.339	12.97
Adadelta	0.052	42.18	0.496	7.012	0.084	12.99
AMSGrad	0.062	39.11	0.467	6.894	0.275	12.85
RMSprop	0.063	38.89	0.516	6.839	0.080	12.41
SGD	0.051	45.91	0.671	7.343	0.093	13.65
Ours	0.048	47.40	0.847	7.405	0.284	15.90

**Table 4:** Segmentation performance (mIoU) of visible, infrared, and fused images in the MSRS dataset. RED indicates the best result, and BLUE represents the second-best result.

Method	Background	Car	Person	Bike	Curve	Car Stop	Cuadrail	Color Tone	Bump	mIoU
Visible	98.26	89.03	59.94	70.00	60.69	71.43	77.90	63.42	75.31	74.00
Infrared	98.24	87.33	70.46	69.23	58.74	68.85	65.57	56.93	72.72	72.01
GANMcC	98.47	89.26	72.11	71.74	62.71	72.94	74.05	63.26	77.42	75.77
U2Fusion	98.49	89.78	72.93	71.96	62.84	70.95	79.25	63.59	77.32	76.35
SDNet	98.52	90.43	73.41	71.61	63.68	75.59	85.25	61.82	81.41	76.11
Adadelta	95.44	58.06	49.53	25.69	9.55	26.92	45.71	35.7	13.57	40.02
AMSGrad	98.46	90.72	66.24	68.75	56.73	73.24	83.14	66.55	80.4	76.02
RMSprop	98.44	90.54	65.37	68.70	56.13	72.76	83.41	66.46	80.42	76.80
SeaFusion	98.45	90.62	65.47	68.81	56.79	73.16	82.99	66.56	80.11	75.88
Ours	98.62	91.06	75.61	71.98	63.92	74.84	77.89	67.31	77.13	77.59

**Table 5:** The table presents the experimental results of training using different methods in different datasets.

Dataset	Method	SD	PSNR	MSE	MI	SDC	SSIM
TNO	SDCFusion	45.35	61.48	0.046	2.236	1.866	0.490
TNO	Ours1	45.37	61.99	0.041	2.387	1.836	0.494
TNO	Ours2	45.82	62.03	0.040	2.365	1.850	0.497
RoadScene	SDCFusion	44.47	58.66	0.088	2.620	1.778	0.398
RoadScene	Ours1	47.87	58.99	0.081	3.340	1.647	0.410
RoadScene	Ours2	50.43	58.82	0.085	3.187	1.620	0.410
MSRS	SDCFusion	42.37	64.37	0.348	4.442	1.665	0.497
MSRS	Ours1	42.28	64.41	0.345	4.869	1.601	0.493
MSRS	Ours2	42.79	64.26	0.336	4.944	1.586	0.497

**Table 6:** A comparison of the performances of different detection models on various metrics in the MSRS dataset.

Model	MSE	VIF	PSNR	AG	CC	SCD	SF	Nabf	alpha	beta	gamma
HC-SPA	0.034	0.843	64.49	3.286	0.674	1.717	8.769	0.003	2.0	0.90	5.0
HC-SPA1	0.039	0.942	63.84	3.031	0.604	1.472	9.400	0.009	0.5	0.90	2.0
HC-SPA2	0.037	0.927	64.08	2.987	0.606	1.490	9.268	0.009	1.0	0.90	10.0
HC-SPA3	0.037	0.945	64.11	2.995	0.608	1.516	9.309	0.009	1.0	0.95	10.0
HC-SPA4	0.041	0.909	63.75	3.079	0.595	1.394	9.560	0.009	1.0	0.90	20.0
HC-SPA5	0.068	0.027	49.83	0.779	0.131	0.249	1.975	0.016	2.0	0.90	10.0
HC-SPA6	0.042	0.934	63.68	3.075	0.602	1.434	9.460	0.008	2.0	0.95	20.0

## Data Availability

The MSRS (Multi-Spectral Remote Sensing) dataset, which provides paired visible and infrared images for multi-modal image fusion research, is publicly available for download from its associated GitHub repository: https://github.com/Linfeng-Shan/MSRS_Fusion (accessed on 15 May 2024). The TNO and RoadScene datasets, comprising co-registered RGB and thermal infrared imagery crucial for evaluating multi-modal remote sensing tasks, can be accessed via their respective repositories: TNO dataset at https://figshare.com/articles/dataset/TNO_Image_Fusion_Dataset/1008029 and RoadScene dataset at https://github.com/huangjunjie2017/RoadScene (accessed on 15 May 2024). All data used in this study are open-source and freely accessible for academic and research purposes. The original contributions presented in this study are included in the article. Further inquiries can be directed to the corresponding author.
